# Assessment of Different Experimental Setups to Determine Viral Filtration Efficiency of Face Masks

**DOI:** 10.3390/ijerph192215353

**Published:** 2022-11-21

**Authors:** Arijana Filipić, Katja Fric, Maja Ravnikar, Polona Kogovšek

**Affiliations:** Department of Biotechnology and Systems Biology, National Institute of Biology, Večna pot 111, 1000 Ljubljana, Slovenia

**Keywords:** Face masks, Virus filtration efficiency, Bacterial filtration efficiency, EN 14683:2019+AC:2019, Air sampler

## Abstract

As a result of the COVID-19 pandemic, many new materials and masks came onto the market. To determine their suitability, several standards specify which properties to test, including bacterial filtration efficiency (BFE), while none describe how to determine viral filtration efficiency (VFE), a property that is particularly important in times of pandemic. Therefore, we focused our research on evaluating the suitability and efficiency of different systems for determining VFE. Here, we evaluated the VFE of 6 mask types (e.g., a surgical mask, a respirator, material for mask production, and cloth masks) with different filtration efficiencies in four experimental setups and compared the results with BFE results. The study included 17 BFE and 22 VFE experiments with 73 and 81 mask samples tested, respectively. We have shown that the masks tested had high VFE (>99% for surgical masks and respirators, ≥98% for material, and 87–97% for cloth masks) and that all experimental setups provided highly reproducible and reliable VFE results (coefficient of variation < 6%). Therefore, the VFE tests described in this study can be integrated into existing standards for mask testing.

## 1. Introduction

The COVID-19 pandemic, caused by severe acute respiratory syndrome coronavirus 2 (SARS-CoV-2), has drastically changed our daily lives. Even though more than two years have passed since the pandemic began, some questions still have not been fully answered. One of them is the exact mode of SARS-CoV-2 transmission [1]. It is known that close proximity to an infected person presents the highest risk of contracting the virus through inhalation or direct deposition of infected droplets on mucous membranes (direct transmission), but it is not entirely clear if the larger droplets or, the smaller droplets/aerosols are mainly responsible for virus transmission in such cases [2]. The importance of airborne transmission with aerosols over longer distances (>2 m; indirect transmission) has also become clear, especially in closed, crowded spaces, with inadequate ventilation [2,3]. Indeed, a growing body of evidence points to the importance of such transmission [4], which is also supported by findings on other respiratory pathogens [5]. Furthermore, it is known that SARS-CoV-2 can remain infectious on different surfaces for days [6,7,8,9]. However, these studies were conducted under laboratory conditions that are very different from a real-world environment. It is estimated that the chance of becoming infected for each contact with surfaces is less than 1 in 10,000 [10]. Therefore, this indirect transmission has been shown to have the least impact on the spread of SARS-CoV-2 [2]. The ambiguity regarding the most important modes of transmission is not specific only to SARS-CoV-2 but also to other respiratory pathogens [5].

Larger droplets are generally considered heavier particles, which quickly fall on the ground before evaporating completely. Therefore, they are only present in close proximity to the infected person (up to 2 m) [11]. On the other hand, aerosols are smaller, lighter droplets that remain airborne for longer periods of time, even hours [11], and can travel farther than 2 m [12]. The cut-off between larger droplets and aerosols is often considered to be 5 μm (even by major health agencies including the World Health Organization and the Centers for Disease Control and Prevention [13,14]), but some studies also indicate otherwise (i.e., the cut-off should be set higher), as discussed in Wang et al., 2021 [11]. The spread of aerosols and their contents (like viruses) in the air depends on environmental factors such as relative humidity and temperature [11]. Aerosols are particularly important for spreading viruses, because they can linger in the air and cause infections over longer distances and usually contain more viruses and can invade the lower respiratory tract [11]. It has been shown that the viral load of SARS-CoV-2 is higher and persists longer in the lower respiratory tract than in the upper respiratory tract [15,16]. 

Regardless of which particles are the most important in the transmission of SARS-CoV-2, the virus can be transmitted with droplets of different sizes produced by symptomatic or asymptomatic individuals when coughing, sneezing, singing, or even just talking and breathing [11]. Therefore, the transmission of pathogens must be stopped at the source (i.e., at the mouth), and the proper use of masks is crucial. 

The most commonly used masks to protect against SARS-CoV-2 include respirators, surgical or medical masks, and cloth masks [17]. Respirators are regulated as respiratory protection devices and are designed to meet more demanding performance criteria. They fit closely to the face of the wearer and provide the best protection for and from the wearer. They are multi-layered (often four-layered), made of non-woven fibrous materials such as polypropylene, and allow for high particle filtration efficiency (PFE). PFE is determined with aerosolized NaCl with particle size distribution between 0.02 µm and 2 µm and is one of the parameters used to classify masks into different categories, i.e., filtering facepieces FFP1, FFP2, and FFP3 with the PFE ≥80, ≥94 and ≥99, respectively [18] Surgical masks are also multi-layered (often three-layered) and are made of the similar materials as respirators, often polypropylene. Bacterial filtration efficiency (BFE), determined with particles ranging in size from 0.65 µm to 7 µm, is one of the parameters that classify surgical masks into Type I or II, with a BFE of ≥95 or ≥98, respectively [19]. Due to their high filtration performance, they can provide a high level of protection. However, they are not as fitted as respirators, so the user and the environment are not as well protected as respirators because the gaps between the mask and the user can serve as a pathway for infection [17,20]. Both respirators and surgical masks are usually intended for single use and undergo rigorous testing before they reach the market, whereas cloth masks do not [17]. There are many advantages to using cloth masks, especially from an environmental standpoint, as they can be reused. They can be made of different materials, such as cotton or silk, with a different number of layers. Their filtration efficiency can vary drastically depending on the characteristics of the mask, such as the type of material, the number of layers, the thread count, and the fit of the mask [21]. Although most cloth masks do not provide as high a level of protection for and from the wearer compared to some surgical masks or respirators (due to often lower filtration efficiency and inadequate fit), they can provide some level of protection [20]. 

To ensure the quality of masks and determine their efficacy, it is important to test various parameters, including filtration efficiency (FE). In Europe, the standard for surgical masks EN 14683:2019+AC:2019 [19] specifies the properties of surgical masks that must be tested. This includes bacterial filtration efficiency (BFE) using *Staphylococcus aureus*. However, there is no standardized method for testing viral filtration efficiency (VFE). Nevertheless, some laboratories are already performing such tests [22]. In terms of research, only a few studies are available on determining the VFE of different masks [23,24,25], reporting a limited amount of experimental data. Therefore, there is a need for a study that evaluates the suitability and efficiency of different systems for determining the VFE. We developed four different experimental setups for VFE testing based on the standard EN 14683:2019+AC:2019 using bacteriophage MS2 and determined their applicability on six types of masks (e.g., a surgical mask, a respirator, materials for mask production, and cloth masks) made from various materials. In comparison, we determined the BFE of these masks in two different experimental setups. This allowed us to assess the performance of a non-standardized (VFE) method in relation to a standardized (BFE) method that has been used for years. Furthermore, we evaluated the suitability of bacteriophage MS2 as a model virus for different experimental setups of the VFE [24]. Thus, this study included numerous experiments, namely 17 BFE and 22 VFE experiments with 73 and 81 mask samples, respectively. It was shown that the results of all experimental setups used for VFE determination were repeatable and reliable. Therefore, the developed system for VFE testing could be implemented in the existing standards for mask testing, if needed. In addition, the results may serve as a good starting point for other research groups working on personal protection and preventing pathogen transmission.

## 2. Materials and Methods

### 2.1. Types of Masks Tested

**Figure 1:**
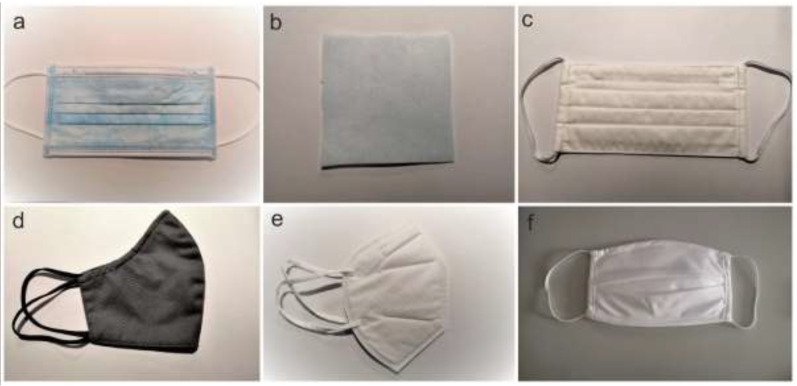
Six different mask samples were used for assessment of different experimental setups used for VFE and BFE tests: mask sample A was a three-layer polypropylene surgical mask classified as Type II, according to the standard EN 14683:2019+AC:2019 (**a**), mask sample B was a three–layer polypropylene material for mask production (**b**), mask samples C and F were reusable two-layer masks made only of woven cotton (**c**,**f**), mask sample D was a reusable mask made of woven cotton, polyester (outer layer), and polypropylene (inner layer) (**d**), whereas masks sample E was a five-layer respirator, classified as FFP2 according to EN 149:2001+A1:2009, made of nonwoven, meltblown, and cotton fabric (**e**).

We tested six types of masks or materials for masks (henceforth referred to as mask samples) that were either purchased or homemade and thus not officially on the market (Figure 1). Two of the mask samples were produced in accordance with standards, i.e., a three-layer polypropylene surgical mask classified as Type II (EN 14683:2019+AC:2019 [19]) (A) and a five-layer respirator classified as FFP2 (EN 149:2001+A1:2009 [18]) made of nonwoven, meltblown and cotton fabric (E). A local mask manufacturer provided a three-layer polypropylene material for mask production (B). A homemade reusable two-layer woven cotton mask (C) and a reusable two-layer mask with an outer layer of woven cotton and polyester and an inner layer of polypropylene (D) were provided by small private mask producers. A reusable two-layer woven cotton mask (F) was purchased in a local pharmacy shop. Each mask was conditioned in a chamber with 250 g of KCl and 0.5 L of dH_2_O for a minimum of 4 h at 21 °C ± 5 °C and relative humidity of 85% RH ± 5% RH prior to testing.

### 2.2. Experimental Setups and Performance of FEs

**Figure 2:**
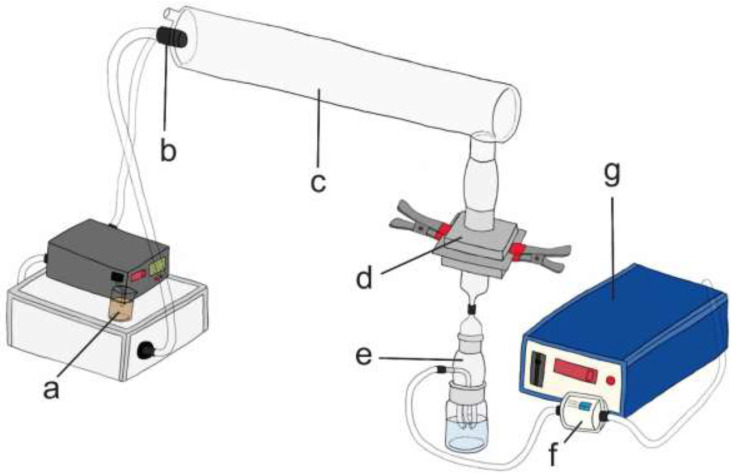
Experimental setup for determination of bacterial and viral filtration efficiency. Bacterial/viral sample (**a**), nebulizer (**b**), glass chamber (**c**), two-piece component into which the mask was tightly clamped (**d**), sampler (**e**); a representative impinger is shown in this figure, whereas we used two types of impinger or the Andersen sampler), HEPA filter (**f**) and the vacuum pump (**g**).

A system constructed for the determination of BFE in accordance with EN 14683:2019+AC:2019 standard was used for all experiments (Table 1, Figure 2), differing mainly by the type of sampler, airflow, and, in the case of the experimental setup VI, also the pump. Two experimental setups were used to determine the BFE. In the first, bacteria were collected directly on the plates using a 6-stage Andersen sampler (Honri Airclean Technology, Suzhou, China), while in the second, they were collected in a liquid medium in the impinger type 1 (Table 1). To determine the VFE, four experimental setups were used. Viruses were collected with an Andersen sampler or with type 1 or type 2 impinger (Table 1). The main difference between the impingers was their size, the radius of the top of the impinger, the complexity of the hooks, and manufacturing as the impinger type 1 were homemade, larger, with a narrower top of the impinger and simpler hooks, while the impinger type 2 was standardized BioSampler (SKC, Dorset, UK). In experimental setups I to V, vacuum pump from 6-stage Andersen sampler was used, while in experimental setup VI BioLite+ Pump (SKC, Dorset, UK)was combined with impinge type 2. 

First, 13 μL of a liquid sample containing either a bacterium (*Staphylococcus aureus,* ATCC 6538) or a virus (bacteriophage MS2, ATCC 15597-B1) (Figure 2a) was aerosolized (Figure 2b), producing droplets and aerosols of different sizes, with an average value of 3.1 ± 0.3 μm (supplementary Tables S1–S6). The generated droplets and aerosols were mixed with ambient air at a flow rate of 28.3 L/min (this mimics a respiratory flow rate; setups I and III), 31.2 L/min (setups II and IV), 10.3 L/min (setup V), or ~6.1 L/min (setup VI). Other flow rates were chosen because they allowed the optimal collection of a model microorganism in each experimental setup. After the 1-minute aerosolization, air flowed through the system for another minute (for BFE and VFE in Andersen sampler) or two minutes (for other setups of VFE) at the same flow rate as just described. The airflow carried droplets and aerosols through the glass chamber with standardized dimensions (Figure 2c), where they reached the two-piece component in which the mask was tightly clamped, with the inside of the mask facing upward (Figure 2d). In the case of the positive controls (PCs), there was no mask in the two-piece component. Droplets and aerosols were then collected in a 6-stage Andersen sampler or a type 1 or 2 impinger (Figure 2e). In each experiment, the first PC was performed first, then 3–5 mask subsamples were tested, followed by the second PC. After each experiment, a negative control (NC; air flowing for 2 or 3 min without bacteria or viruses) was performed. The pressure was always maintained at 0.35 bar. In the end, the system was first cleaned by the aerosolization of 70 % ethanol or 4.9 % hydrogen peroxide and then Milli-Q water, and the equipment was washed, autoclaved and/or sterilized with the UV light.

Most of the experiments were performed with mask samples A and B. This allowed us to compare the results of different experimental setups directly and to determine the reliability and repeatability of the VFE tests. Other masks were included in the experiments to investigate whether the developed VFE tests are suitable for evaluating the FE of masks made of different materials and different qualities and to obtain a more comprehensive overview of the FE of different masks. It also allowed us to determine whether MS2 is a suitable virus for VFE tests performed in different experimental setups. In this regard, the repeatability of the FE values, the droplets’ average diameter, and the virus concentrations’ stability in the positive controls were considered the most important characteristics determining the suitability of the model microorganism.

#### 2.2.1. BFE

##### BFE with 6-Stage Andersen Sampler (Experimental Setup I)

In the experimental setup I, a 6-stage Andersen sampler was used, 11 experiments were performed, and a total of 45 mask subsamples were tested (Table 1, supplementary Table S1). BFE was determined according to the standard EN 14683:2019+AC:2019 [19], described in Košir et al., 2022 [26], with small modifications, including a wider range of average bacterial concentration in positive controls that was considered to be appropriate, i.e., 1.28 × 10^3^–3.07 × 10^3^ colony forming units (CFU)/test. For each PC, mask sample, or NC, bacteria were collected on six plates in a 6-stage Andersen sampler. Each stage had 400 openings of different diameters, with the largest at the top (first stage) and the smallest at the bottom (sixth stage) to mimic the flow of inhaled particles in the human respiratory system (the diameters of the openings on the same stage were the same) [26]. Plates were prepared from 40 g/L tryptic soy agar (TSA) (Fluka). After the experiments, plates were incubated overnight at 37 °C, the colonies were counted, and the CFU was determined, considering the positive hole correction [26,27]. The final BFE was calculated as described in the section Calculation of BFE and VFE. The mean particle size was calculated as described in EN 14683:2019+AC:2019 [19] and Košir et al., 2022 [26].

##### BFE with Impinger Type 1 (Experimental Setup II)

In experimental setup II, where the Andersen sampler was replaced by the impinger type 1, 6 experiments were conducted, and 28 mask subsamples were tested (Table 1, supplementary Table S2). The impinger contained 30 mL of peptone water prepared from 10 g/L Bacto peptone (DB) and 5 g/L NaCl (Merck). A new glass cup, i.e., the lower sampling part of the impinger, was used for every subsample (e.g., PC, mask subsample, or NC). At the end of each experiment, 100 μL of undiluted or diluted peptone water from each subsample was spread on two TSA plates. Plates were incubated overnight at 37 °C, and the bacterial colonies were counted. Bacterial concentrations (CFU/mL) were determined considering bacterial dilutions and plating volumes. The final BFE was calculated as described in the section Calculation of BFE and VFE. 

#### 2.2.2. VFE

##### VFE with Andersen Sampler (Experimental Setup III)

In experimental setup III, the experiments were performed, and the final VFE was calculated in the same way as in the experiments for BFE, experimental setup I. The only difference was the type of plates used. Here, the mixture of 5 mL of melted ‘TSB top agar’ and 100 µL of *Escherichia coli* in logarithmic phase was poured onto ‘TSB agar’ plates (explained in the section Double-layer plaque assay), and the plates were allowed to harden. Together, four experiments were conducted, and 12 mask subsamples were tested (Table 1, supplementary Table S3). 

##### VFE with Impingers Type 1 and 2 (Experimental Setups IV, V and VI)

In experimental setup IV, impinger type 1 was used, while in setups V and VI, impinger type 2 was used. In addition, a different vacuum pump was used in setup VI. In experimental setup IV, six experiments with a total of 26 mask subsamples were performed (supplementary Table S4); in setup V, there were nine experiments with 34 mask subsamples (supplementary Table S5); and in the setup V, there were three experiments with nine mask subsamples (supplementary Table S6) (Table 1). In experimental setup IV, viruses were collected in 30 ml of peptone water (it was prepared in the same way as for BFE), while in setups V and VI, they were collected in 10 mL of it. A new glass cup was used for each subsample (e.g., PC, mask subsample, or NC). At the end of the experiment, appropriate dilutions of the peptone water were prepared, and the virus concentrations were determined using double-layer plaque assay, and the final VFE was determined as described in the section Calculation of BFE and VFE.

##### Double-Layer Plaque Assay

Three media were used for double-layer plaque assay (DAL), e.g., ‘TSB agar’, ‘TSB top agar,’ and ‘liquid TSB.’ ‘TSB agar’ prepared from 30 g/L TSB and 15 g/L Bacto agar (BD) was used for agar plates. ‘TSB top agar’ was prepared in the same way, except that 7 g of agar was added and was used as the top layer in this assay. ‘Liquid TSB’ used for cultivation of *E. coli* CB390 [28] was prepared from 30 g TSB/L. All media contained 1.93 g/L MgCl_2_ × 6H_2_O (Duchefa Biochemie) and 100 mg/L ampicillin (Sigma-Aldrich). 

DAL was performed by adding 0.1 mL of *E. coli* in the logarithmic phase (prepared by inoculating 5 mL of ‘liquid TSB’ with 0.2 mL of ~19 h old bacterial culture followed by 3 h incubation at 37 °C and 230 rpm) and 0.25 mL of undiluted or diluted peptone water with viruses to ~5 mL of melted ‘TSB top agar’ in 15 mL glass tubes. This was then mixed thoroughly and poured onto ‘TSB agar’ plates. Each virus dilution was prepared in duplicates or triplicates. After overnight incubation at 37 °C, the number of plaques was counted, and the virus concentrations (plaque forming units, PFU/mL) were calculated considering virus dilutions and plating volumes.

### 2.3. Calculation of BFE and VFE

The first step in determining BFE or VFE in each experiment was to calculate the average value of the two PCs (the first was performed at the beginning of each experiment, the second after 3–5 mask subsamples). This value was then used to calculate BFE or VFE for each mask subsample according to Equation (1):

(1) $$\mathrm{BFE},\mathrm{VFE}\;\left(SB\right)\left(\%\right)=\frac{Cpc-Csb}{Cpc}\times \;100$$

where *Cpc* is the average bacterial/viral concentration of the two PCs and *Csb* is the bacterial/viral concentration in the mask subsample. 

The final BFE or VFE of the same experimental setup was then calculated as the average value of all the subsamples of one mask sample according to Equation (2):

(2) BFE,VFE (%)=average[BFE,VFE (SBs)]

## 3. Results and Discussion

In the absence of standardized methods and research data for determining the VFE of masks, the present study focused on the evaluation of four different experimental setups that can be used for this purpose. In parallel, BFE was determined on the same mask samples according to the standardized method EN 14683:2019+AC:2019 and its modified version. This allowed a direct comparison and assessment of the standardized and non-standardized methods, which further facilitated the evaluation of the quality of VFE tests as well as the suitability of MS2 as a model virus for the VFE test in various experimental setups.

### 3.1. Evaluation of the Experimental Setups 

Initially, five or six different experimental setups (including both BFE and VFE) were tested on two mask samples, A and B, made of three-layer polypropylene, to compare and assess each of the experimental setups. Three to five subsamples of each mask sample were tested in 3–4 independent repetitions, each time giving similar FE results (supplementary Tables S1–S6), indicating high reproducibility of the developed test systems (coefficient of variation, CV, ≤1%) (Table 2). This was also confirmed by the generation of droplets of a similar average diameter of 3.1 μm ± 0.3 μm in the experiments with the Andersen sampler, along with the maintenance of stable bacterial and viral concentrations in the PCs of the same experimental setup (supplementary Tables S1–S6).

Interestingly, the results show that the average BFE and VFE are slightly lower when using the Andersen sampler than when collecting bacteria and viruses in impingers (Table 3). This could be linked to the initial bacterial and viral concentrations, which were lower in experimental setups with the Andersen sampler and up to 3.07 × 10^3^ CFU or PFU (supplementary Tables S1–S6). It is very important to use the correct initial concentration in the Andersen sampler, as the plates can become saturated with microorganisms, preventing an accurate determination of FE. Hence, the statistical correction, i.e., the positive hole correction [27], is applied to determine CFU and PFU, which anticipates that more than one bacteria or virus can pass through each hole of an individual stage (representative plates for BFE and VFE tests in Andersen sampler are shown in supplementary Figure S1). The determined concentration is thus estimated and can differ from the actual concentration determined by classical growing and counting CFU and PFU. Since the VFE values obtained with the Andersen sampler are, on average lower than the VFE values in the impingers, the experimental setup with the Andersen sampler presents a safer choice, as when it comes to protective equipment, it is better to underestimate the FE and test the “worst case” filtration efficiency than to overestimate it [29]. Moreover, working with this sampler allows us to determine the average droplet size and work with airflows corresponding to respiration. In addition, a single Andersen sampler is sufficient for all subsamples, unlike impingers, which require a new glass cup for each subsample. In addition, the plates are transferred from the Andersen sampler directly to the incubator without processing as with impingers.

On the other hand, if it is necessary to work with higher initial concentrations of viruses or if the samples require additional processing and testing for other properties, then impingers are a way to go. However, the type of impinger and pump must be selected based on the desired airflow and considering the practicality of the experimental setup. In our opinion (from the experimental setups that used impingers), the combination in experimental setup V worked the best. A commercial laboratory practice also supports our conclusion, as they use impinger only to determine VFE with an increased challenge, i.e., when the initial viral concentration is higher than 3.3 × 10^3^ PFU/test, while otherwise using Andersen sampler [22]. They, however, do it in combination with phix 174 as a model virus. Only a few other groups have worked on determining VFE in a similar setup. They either worked with MS2 [24] or phix 174 [25], which they sampled using the Andersen sampler. In addition, a completely different experimental setup was also developed to determine VFE, using the mannequin head with an aerosol source simulator and a SARS-CoV- 2 pseudovirus as a model virus [23].

Several other factors are known to affect the FE of masks, including airflow [25]. Higher airflow decreases FE, likely due to the shorter time for droplets to diffuse or interact with the electrostatically charged fibers [30]. In the experimental setups with the Andersen sampler (I and III) and the homemade impinger (II and IV), the flow rate was similar (28.3 L/min vs. 31.2 L/min), while much lower flow rates were measured with type II impinger (V and VI) (10.3 and ~6.1 L/min, respectively). Since the FE results of the same mask sample were similar, regardless of airflow, they indicate that flow rate is not that crucial in VFE tests. This is supported by the fact that the main filtration mechanisms for particles ranging from 0.65 to 7 μm are interception, and inertial separation, followed by gravitational settling and possibly also by electrostatic attraction, depending on the filter material and charge of the droplets produced. These could allow particles to settle on the mask so they would not be affected by the higher flow rates [31].

In addition to mask samples A and B, four other mask samples were used. This enabled us to evaluate if different experimental setups for VFE testing were suitable for testing masks of different quality and FE. It also helped in the final determination of whether MS2 is an appropriate viral model for VFE testing. As was the case for mask samples A and B, the results obtained for the other masks also indicate the robustness, reliability, and repeatability of the experimental procedures developed, as confirmed by the FE results, generation of droplets of similar average diameter along with the maintenance of stable bacterial and viral concentrations in the positive controls of the same experimental setup (supplementary Tables S1–S5). In addition, the CVs for mask samples C–F were also low and, as expected, higher for the masks with the lower FE (Table 2). Therefore, the results obtained with the six different mask samples indicate that MS2 is a suitable viral model for VFE testing for different experimental setups and that VFE testing can be performed for masks made of different materials and with different FEs.

**Figure 3:**
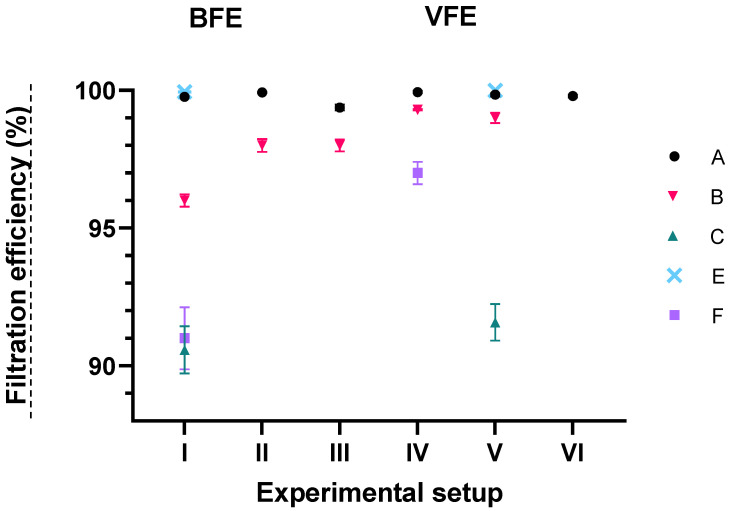
Average filtration efficiencies (bacterial, BFE, and viral, VFE) and their standard errors for each mask sample. BFE and VFE values were calculated from all subsamples of the same experimental setup for an individual mask sample. Results for mask D were not included on the graph due to their lower efficiency.

Despite some observed differences, the average VFE values of the same mask sample are similar regardless of the experimental setup and are comparable to BFE results (Figure 3, Table 3). This is not surprising since the same nebulizer was used in all experiments, producing droplets and aerosols of the same size, with diameters large enough to contain either bacteria (*S. aureus* has a diameter of 0.5–1 μm) or viruses (MS2 has a diameter of about 27 nm). A similar observation was also made by Rengasamy et al., 2017 [25] when they compared BFE and VFE values for the same masks. 

We have shown that all experimental setups tested for the determination of VFE can be sufficient and that the decision of which experimental setup to choose depends on several factors, as described above. Therefore, this study can serve as a great foundation for implementing VFE testing into existing standards for mask testing. To further improve VFE testing, evaluating the FE of masks for the aerosols with the smaller average sizes would be beneficial, as this would provide insight into the FE of masks for small aerosols in which viruses can also reside and spread. This was demonstrated by Santarpia et al., 2021 [32], who found intact SARS-CoV-2 viruses in droplets with an average size between 0.60 and 0.80 μm, which is also consistent with studies conducted prior to the COVID-19 pandemic [33]. Although smaller droplets contain less viruses and therefore pose less of a threat than larger particles, which can contain a higher viral load, they can still present a risk of infection, especially if produced predominantly [34].

### 3.2. Determination of Filtration Efficiency of Masks

As expected, mask sample A, classified as a Type II surgical mask (EN 14683:2019+AC:2019), resulted in BFE and VFE above 99% in all experimental setups (Table 3, Figure 3). A similarly high FE was also obtained for mask sample B, with some observed differences between the experimental setups, which could classify the final product, i.e., surgical masks made from this material, as Type I or II. Knowing that the characteristics of the material can vary and depend on several process parameters [35], the determined differences in FE could be due to the fact that three separate layers of polypropylene were manually assembled for testing, whereas for the final product, all three materials are pressed together. Mask sample E also had a very high FE value ≥ 99.9%, which was expected considering that this mask sample was an FFP2 respirator, although a different standard is normally used to determine the FE of respirators. We also tested the BFE and VFE of three reusable cloth mask samples, C, D, and F. Mask samples C and F, both made of two layers of woven cotton, had an FE of >90%. Mask sample D contained polyester and polypropylene in addition to woven cotton and had lower BFE and VFE of 79 and 87%, respectively. It is known that woven cotton can ensure high FE, but it depends on the number of layers and thread count, as they assure physical filtration. On the other hand, materials like polyester have moderate electrostatic discharge, which is better for the filtration of smaller aerosols (<300 nm) [21]. Since we produced droplets with an average size of 3 µm, we do not know if the FE of these masks would be better for smaller particles only and whether mask sample D would be superior in filtering such particles compared to the other two cloth masks.

Similar observations on the FEs of different masks were made by other groups, which found high FEs (either BFE, VFE, or PFE) in surgical masks and respirators, while cloth masks had different FEs, which in some cases were quite high, above 90% [23,24,25,36]. Caution should be exercised in interpreting these results because the fit of masks is usually not taken into account when FE is determined, and therefore the protection provided by the masks does not necessarily correspond to the measured FE. For example, the BFE systems described in the standards and the VFE systems based on them usually test the FE of one area of the mask (in our case, it was 8 × 8 cm^2^ for respirators and 10 × 10 cm^2^ for other masks) that is tightly clamped so that the air carrying bacteria/viruses can only pass through the material. In reality, various masks, especially cloth masks, do not always fit tightly against the wearer’s face, and these openings can serve as a transmission route for various pathogens. Therefore, in addition to FE, another important property of the mask is fit because the same mask can protect differently depending on the fit; the better the fit, the better the protection [37]. Breathability is another characteristic to consider when discussing mask protection. If too low, it does not only cause discomfort for the wearer, as it interferes with normal breathing but also results in lower FE, as it pushes air-containing pathogens into the gaps between the mask and the wearer instead of filtering the material [38]. Thus, it is not surprising that respirators (which undergo total inward leakage testing) are most effective in containing the spread of SARS-CoV-2 [39]. Because of the preventive measures taken in response to the COVID -19 pandemic, including mask usage and social distancing, it is difficult to find controlled studies that have determined the effectiveness of masks against SARS-CoV-2. However, it has been shown that intrahousehold transmission was significantly reduced when household members wore masks before the onset of symptoms [40]. Another study on the use of face masks in indoor spaces also confirmed that wearing masks reduced the risk of infection with SARS-CoV-2 and that respirators were best, followed by surgical masks [41]. It has also been shown that countries where mask use was encouraged had lower mortality rates from the SARS-CoV-2 virus [42] and that the incidence of infection was lower in areas where universal masking was common [43]. As Cheng et al. (2021) [44] noted, most people stay in environments where the prevalence of droplets and aerosols containing the SARS-CoV-2 virus is low, so all types of masks can help prevent the spread of the virus. However, in environments with higher droplet exposure, such as hospitals, it is recommended to use masks with high FE and good fit. All of these studies suggest that face masks, when worn properly, are a very important part of the contingency plan to prevent the spread of respiratory pathogens such as SARS-CoV-2, which was also confirmed in several studies evaluated by the CDC [45]. The type of mask to choose for protection depends on several factors, such as frequency of contact with infected persons, length of time spent in poorly ventilated enclosed spaces, and the state of the immune system.

## Figures and Tables

**Table 1:** Number of experiments in different experimental setups for each mask sample. Each experiment included 3–5 mask subsamples (supplementary Tables S1–S6). BFE, bacterial filtration efficiency; VFE, viral filtration efficiency; n.t., not tested.

		Number of Experiments					
		BFE		VFE			
Experimental Setup		I	II	III	IV	V	VI
	Sampler Type	Andersen	Impinger Type 1	Andersen	Impinger Type 1	Impinger Type 2	
Mask Sample							+ Different Pump
A		3	3	2	3	3	3
B		4	3	2	2	3	n.t.
C		1	n.t.	n.t.	1	n.t.	n.t.
D		1	n.t.	n.t.	n.t.	1	n.t.
E		1	n.t.	n.t.	n.t.	1	n.t.
F		1	n.t.	n.t.	n.t.	1	n.t.

**Table 2:** Reproducibility of bacterial (BFE) or viral (VFE) filtration efficiency determined with different experimental setups (BFE: setups I and II; VFE: setups III–VI). The coefficient of variation (%) was calculated from all subsamples of each mask sample in the same experimental setup (A–F).

Mask Sample	Experimental Setup	Coefficient of Variation (%)
A	I	0.16
	II	0.07
	III	0.26
	IV	0.15
	V	0.17
	VI	0.14
B	I	1.00
	II	0.94
	III	0.48
	IV	0.09
	V	0.61
C	I	2.14
	IV	0.73
D	I	5.34
	V	3.15
E	I	0.06
	V	0.002
F	I	2.11
	V	1.63

**Table 3:** Average bacterial (BFE) and viral filtration efficiency (VFE).

Experimental Setup	I	II	III	IV	V	VI	Type ^a^
Mask Sample	BFE (%) ^b^		VFE (%) ^b^				
A	99.8	99.9	99.4	99.9	99.8	99.8	II
B	96	98	98	99.3	99	-	I or II
C	91	-	-	-	92	-	NA
D	79	-	-	-	87	-	NA
E	99.9	-	-	-	99.999	-	II
F	91	-	-	97	-	-	NA

BFE and VFE values were calculated from all subsamples of the same experimental setup for an individual mask sample; ^a^ According to EN 14683:2019+AC:2019, Type I and II are determined. This is not applicable (NA) to reusable cloth masks. ^b^ When the BFE and VFE values were between 99% and 100%, more decimal places were included to show the exact filtration efficiency of the mask.

## Supplementary Materials

The following supporting information can be downloaded at: https://www.mdpi.com/article/10.3390/ijerph192215353/s1, Table S1: Results for all mask samples in experimental setup I; Table S2: Results for all mask samples in experimental setup II; Table S3: Results for all mask samples in experimental setup III; Table S4: Results for all mask samples in experimental setup IV; Table S5: Results for all mask samples in experimental setup V; Table S6: Results for all mask samples in experimental setup VI; Figure S1. Plates with Staphylococcus aureus colonies (left) or MS2 plaques (right) after testing bacterial filtration efficiency (BFE) or viral filtration efficiency (VFE) in the Andersen sampler (experimental setups I and III).
