# Supplementary material for: Assessment of Different Experimental Setups to Determine Viral Filtration Efficiency of Face Masks
Source: Int J Environ Res Public Health. 2022 Nov 21;19(22):15353. doi: 10.3390/ijerph192215353 (PMC9690668; doi:10.3390/ijerph192215353)

Supplementary Information

Assessment of different experimental setups to determine viral filtration efficiency of face masks

Arijana Filipić[1], Katja Fric[1], Maja Ravnikar[1], Polona Kogovšek[1]

[1]Department of Biotechnology and Systems Biology, National Institute of Biology, Ljubljana, Slovenia

Supplementary tables S1-S6. Filtration efficiency results for all mask samples in experimental setups I-VI. CFU, colony forming units; PFU, plaque forming units; PC, positive control; NC, negative control; - this measurement was not included in the experiment. When the BFE and VFE values were between 99% and 100%, more decimal places were included to show the exact filtration efficiency of the mask.

Supplementary Table S1. Results for all mask samples in **experimental setup I**

Mask sample	Average concentration of two PCs (CFU)	BFE in subsamples (%)					NC (CFU)	Average mean particle size (µm)
A	2.70E+03	99.93	99.9	99.93	-	-	0	3.1
	2.51E+03	99.4	99.7	99.7	-	-	0	3.1
	2.42E+03	99.9	99.8	99.7	-	-	-	3.0
B	2.80E+03	97	98	97	98	97	0	2.9
	3.06E+03	97	97	97	97	96	0	3.0
	2.92E+03	95	94	96	97	96	0	3.0
	1.28E+03	97	95	97	-	-	1	3.0
C	1.96E+03	92	89	93	-	-	0	3.1
D	2.38E+03	73	76	79	80	86	0	2.9
E	3.07E+03	99.93	99.97	99.8	100	99.97	1	3.0
F	2.41+E03	88	93	91	88	92	0	3.0

Supplementary Table S2. Results for all mask samples in **experimental setup II**

Mask sample	Average concentration of two PCs (CFU/mL)	BFE in subsamples (%)					NC (CFU/mL)
A	4.46E+03	99.8	99.8	99.9	-	-	0
	1.38E+04	99.96	99.9	99.96	99.96	99.96	0
	8.66E+03	99.9	100.0	100.00	99.94	99.94	0
B	1.52E+04	99	99	99,0	99	99,4	0
	7.30E+03	96	98	97	98	97	0
	1.74E+04	98	97	98	98	99	0

Supplementary Table S3. Results for all mask samples in **experimental setup III**

Mask sample	Average concentration of two PCs (PFU)	VFE in subsamples (%)					NC (PFU)	Average mean particle size (µm)
A	2.86E+03	99.6	99.4	99.8	-	-	0	3.4
	2.19E+03	99.2	99	99.4	-	-	0	3.1
B	2.36E+03	98	98	90[a]	-	-	0	3.1
	2.93E+03	99	98	97	-	-	0	3.2

[a] This result was not considered in the calculation of the final VFE because there was likely a problem in the preparation of this mask that influenced the lower VFE. Namely, mask type B is a three-layer material that we had to assemble before each experiment. This VFE proved to be a significant outlier ($P < 0.01$) when compared to other VFE values from the same experimental setup (III) as well as VFE values from all experimental setups (https://www.graphpad.com/quickcalcs/grubbs2/).

Supplementary Table S4. Results for all mask samples in **experimental setup IV**

Mask sample	Average concentration of two PCs (PFU/mL)	VFE in subsamples (%)					NC (PFU/mL)
A	1.17E+05	99.98	100	99.4	-	-	0
	8.57E+04	99.99	99.98	99.98	99.98	99.98	0
	1.21E+05	99.98	99.98	99.98	99.96	99.97	0
B	6.52E+04	99.3	99.3	99.2	99.5	99.3	0
	1.24E+05	99.2	99.3	99.4	99.3	99.3	0
C	1.28E+05	98	96	96	-	-	0

Supplementary Table S5. Results for all mask samples in **experimental setup V**

Mask sample	Average concentration of two PCs (PFU/mL)	VFE in subsamples (%)					NC (PFU/mL)
A	1.35E+06	100	100	99.99	-	-	0
	1.16E+06	99.8	99.92	99.9	-	-	0
	5.41E+05	99.5	99.7	99.7	-	-	0
B	6.58E+05	98	98	98	98	-	10
	1.79E+06	99.93	99.4	99.3	-	-	0
	2.56E+06[a]	99.7	99.1	99.2	-	-	0
D	6.11E+05	83	86	88	91	88	0
E	6.96E+05	99.999	100	99.996	100	99.998	0
F	1.36E+06	91	92	89	94	91	0

[a]This value is only from the first positive control as the second one was not performed during the experiment.

Supplementary Table S6. Results for all mask samples in **experimental setup VI**

Mask sample	Average concentration of two PCs (PFU/mL)	VFE in subsamples (%)					NC (PFU/mL)
A	1.29E+06	99.9	99.8	99.9	-	-	0
	9.36E+05	99.9	99.9	99.9	-	-	0
	3.98E+05	99.8	99.7	99.4	-	-	0

Supplementary Figure S1. Plates with *Staphylococcus aureus* colonies (left) or MS2 plaques (right) after testing bacterial filtration efficiency (BFE) or viral filtration efficiency (VFE) in the Andersen sampler (experimental setups I and III). The observed bacterial/virus pattern follows the pattern of the holes on each stage. The top two rows of plates (top 6 plates) show the positive control plates (from one of the two positive controls), i.e., the plates without a mask, whereas the bottom two rows (bottom 6 plates) show the plates where the bacterial or viral droplets passed through the mask. The mask on the left had a BFE of 97%, while the mask on the right had a VFE of 99.2%.

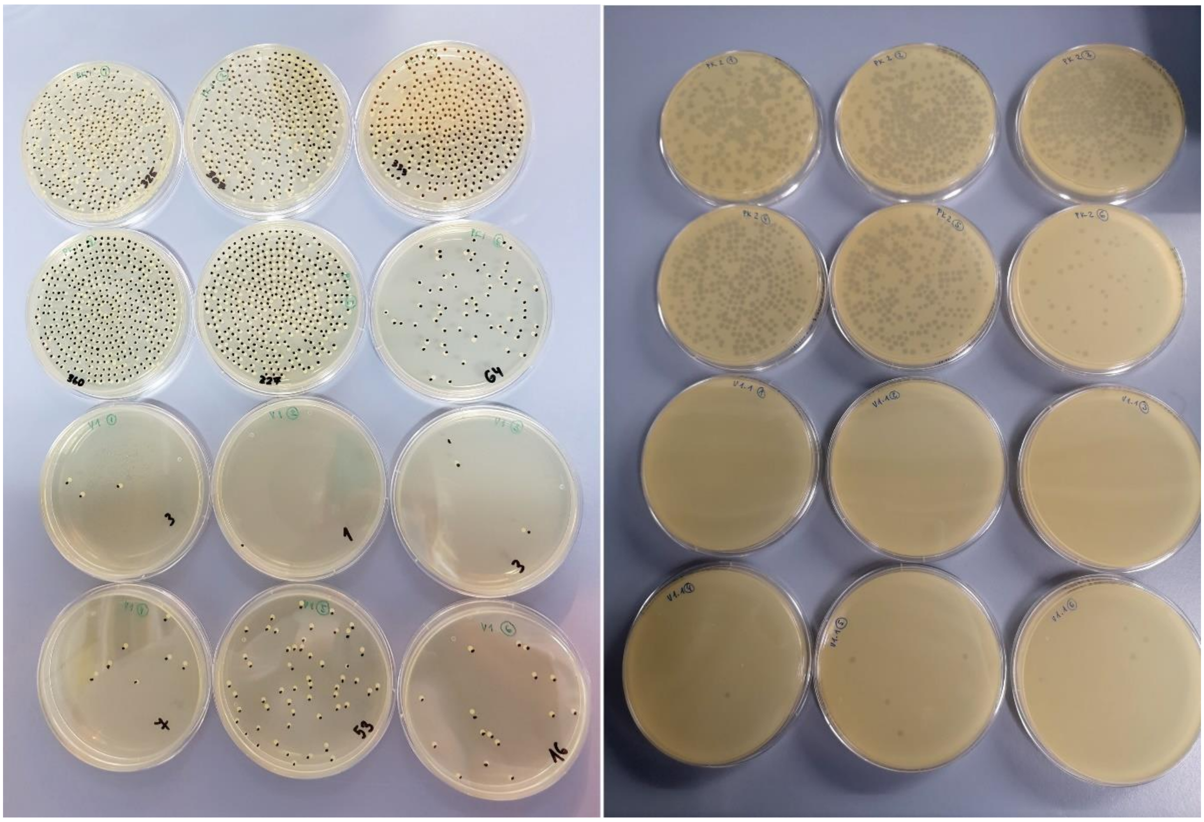

Supplement: Supplementary file 1 [file ijerph-19-15353-s001.zip › ijerph-2007982-supplementary.pdf]
